# The role of psychological strengths in positive life outcomes in adults with ADHD

**DOI:** 10.1017/S0033291725101232

**Published:** 2025-10-06

**Authors:** Luca D. Hargitai, Emma L. M. Laan, Lessa M. Schippers, Lucy A. Livingston, Graeme Fairchild, Punit Shah, Martine Hoogman

**Affiliations:** 1 Department of Psychology, University of Bath, Bath, UK; 2 Department of Psychiatry, Radboud University Medical Center, Donders Institute for Brain, Cognition and Behaviour, Nijmegen, Netherlands; 3 Karakter Child and Adolescent Psychiatry Centre, Nijmegen, Netherlands; 4 Department of Psychology, Institute of Psychiatry, Psychology and Neuroscience, King’s College London, London, UK; 5 Department of Medical Neuroscience, Radboud University Medical Center, Donders Institute for Brain, Cognition and Behaviour, Nijmegen, Netherlands

**Keywords:** attention-deficit/hyperactivity disorder, mental health, psychological strengths, quality of life, wellbeing

## Abstract

**Background:**

Strength-based approaches are increasingly common in neurodevelopmental research, but the positive characteristics that may be features of attention-deficit/hyperactivity disorder (ADHD) remain underexplored. The extent to which people with ADHD recognize and use their personal strengths, and whether these play a role in their life outcomes, is also unknown. Tackling these gaps in the literature, we conducted the first study of self-reported strengths, strengths knowledge, and strengths use in ADHD.

**Methods:**

Adults with (*n* = 200) and without (*n* = 200) ADHD were recruited online and rated their endorsement of 25 putative ADHD-related strengths. Participants also completed self-report measures assessing strengths knowledge, strengths use, subjective wellbeing, quality of life, and mental health. Using both Frequentist and Bayesian methods, we compared the groups and explored the associations of strengths knowledge and use with outcomes across both groups.

**Results:**

The ADHD group endorsed 10 strengths more strongly than the non-ADHD group, including hyperfocus, humor, and creativity, but reported similar endorsement for 14 of the strengths. Adults with and without ADHD did not differ on their strengths knowledge and use but, in both groups, increased strengths knowledge and, to some extent, greater strengths use were associated with better wellbeing, improved quality of life, and fewer mental health symptoms.

**Conclusions:**

We conclude that, while adults with and without ADHD may have both similarities and differences in strengths, interventions that focus on enhancing people’s strength knowledge and promoting the everyday use of their personal strengths could have universal applications to improve wellbeing in adulthood.

## Introduction

Over the past decade, there has been a fast-growing shift toward a strengths-based conceptualization of neurodevelopmental conditions, like autism spectrum disorder (hereafter autism) and attention-deficit/hyperactivity disorder (ADHD), which moves away from traditional, deficit-based models in psychiatry (Fung & Doyle, 2021; Shah & Holmes, 2023). This approach, drawing on the neurodiversity paradigm, seeks to promote positive life outcomes through emphasizing the recognition and use of ‘neurodivergent strengths’ (i.e., psychological qualities and skills that are thought to be heightened or more common in neurodivergent individuals; Huntley et al., 2019; Taylor, Livingston, Clutterbuck, Callan, & Shah, 2023). This strengths-focused approach has been particularly prominent in autism research, where interventions that harness putative autistic strengths – such as attention to detail, logical thinking, adherence to routines, and recognizing patterns – are being widely recommended and applied with the aim of improving autistic people’s quality of life, mental wellbeing, and educational and employment outcomes (Huntley et al., 2019; Lee et al., 2024; Murthi, Chen, Shore, & Patten, 2023; Urbanowicz et al., 2019). Notwithstanding debates on the quality of the historical evidence base (for discussion, see Taylor et al., 2023), there is now a rapidly growing body of research on qualities and skills that can be considered psychological strengths in autism.

There has been far less research on strengths in ADHD, with a narrow focus on creativity (i.e., generating original ideas that offer effective and appropriate solutions to problems; Runco & Jaeger, 2012) and divergent thinking (i.e., the ability to think in unconventional ways; Acar & Runco, 2015). While most of this research points toward a positive association between ADHD and creativity (e.g., Girard-Joyal & Gauthier, 2022; Stolte et al., 2022; White & Shah, 2006, 2011), some studies examining children and adolescents have not found differences in creative ability between ADHD and non-ADHD groups (e.g., Abraham, Windmann, Siefen, Daum, & Güntürkün, 2006; Aliabadi, Davari-Ashtiani, Khademi, & Arabgol, 2016; Healey & Rucklidge, 2005). These disparities may partly stem from broader methodological issues within the quantitative studies examining creativity and divergent thinking in ADHD. As Hoogman, Stolte, Baas, and Kroesbergen (2020) highlight, many studies employ small samples, which lead to underpowered analyses and unreliable results (see Button et al., 2013; Hobson, Poole, Pearson, & Fletcher-Watson, 2022). There is also a widespread failure to account for confounding variables (e.g., education level, socioeconomic status; Castillo-Vergara, Barrios Galleguillos, Jofré Cuello, Alvarez-Marin, & Acuña-Opazo, 2018; Gajda, 2016; Squalli & Wilson, 2014), which may further contribute to the mixed results in the literature. More broadly, it could also be argued that a narrow focus on creativity, without considering other psychological strengths, has done relatively little to advance the study of ADHD-related strengths.

Tackling this limitation, a broader array of potential psychological strengths has emerged from qualitative studies on the lived experiences of adults with ADHD. Beyond creativity and divergent thinking, the ability to multitask, high levels of energy and drive, hyperfocus, adventurousness, a willingness to take risks, and empathy were commonly self-reported strengths in ADHD (Fleischmann & Miller, 2013; Holthe & Langvik, 2017; Mahdi et al., 2017; Sedgwick, Merwood, & Asherson, 2019). Although of great interest, most of these investigations were small in scale and/or sampled only ‘successful’ adults with ADHD (e.g., those with higher education degrees, in long-term relationships and in full-time employment), which makes these findings difficult to generalize to the wider ADHD population. To gain greater insight into the psychological strengths that adults associate with their ADHD, Schippers et al. (2022) recently analyzed rich qualitative data from over 200 adults with ADHD. Creativity, being dynamic, flexibility, socioaffective skills (e.g., empathy, being socially outgoing, being sensitive), and higher-order cognitive skills (e.g., intelligence, being analytical, hyperfocus) emerged as the key domains of psychological strength in ADHD. Some adults also viewed certain core ADHD symptoms as inherent strengths, for example, linking their impulsivity to being spontaneous and their hyperactivity to being energetic and enthusiastic. In line with these findings, recent quantitative evidence from Schippers, Greven, and Hoogman (2024) shows that some psychological strengths self-reported by adults with ADHD, namely, hyperfocus, cognitive flexibility, and sensory processing sensitivity, are also associated with elevated ADHD traits in the general population.

Despite a growing recognition of the skills and traits that may be considered strengths in ADHD, several important questions remain unanswered. First, in the absence of a large, well-powered comparison between adults with and without ADHD, it is unclear whether the skills and characteristics that are assumed to be ‘ADHD strengths’ are indeed more recognized among individuals with ADHD as implied by this term. Second, research to date has focused exclusively on identifying and collating psychological strengths associated with ADHD. Therefore, we currently have no indication of the extent to which adults with ADHD recognize these proposed strengths within themselves (this is termed their ‘strengths knowledge’), and there is no research on how often they utilize these strengths in their daily lives (i.e., their ‘strengths use’). Finally, research in autistic populations indicates that their strengths use and strengths knowledge may be important predictors of better mental health, wellbeing, and quality of life (Taylor et al., 2023). These findings, which have key implications for interventions aimed at improving wellbeing in autism, point toward the need for similar investigations in ADHD.

Building on this recent research, we compared the self-reported psychological strengths of a large, well-matched sample of adults with and without ADHD. We hypothesized that adults with ADHD would report more ADHD-related strengths compared to those without ADHD, with creativity being one of the most highly endorsed strengths in the ADHD group. We further compared the ADHD and non-ADHD groups on their strengths knowledge and use, predicting that adults with ADHD would report lower knowledge and use of their personal strengths than adults without ADHD. Finally, we undertook secondary analyses to examine the extent to which strengths knowledge and use predict quality of life, wellbeing, and mental health in adults with and without ADHD.

## Methods

### Participants

Four-hundred adults from the United Kingdom (200 with and 200 without ADHD) were recruited via Prolific, an online platform that facilitates high-quality data collection from diverse samples, including large-scale research on neurodevelopmental conditions (Douglas, Ewell, & Brauer, 2023; Palan & Schitter, 2018; Peer, Brandimarte, Samat, & Acquisti, 2017; Taylor, Farmer, Livingston, Callan, & Shah, 2022). Given the frequent co-occurrence and overlapping symptom profiles of autism and ADHD (e.g., Antshel & Russo, 2019; Waldren et al., 2024), we used preexisting filters in Prolific to ensure that only people who indicated that they did not have autism and did not suspect they were autistic participated. All participants in the ADHD group were screened to ensure that they (i) self-reported a formal clinical diagnosis of ADHD from a psychiatrist, psychologist or other medically qualified specialist and (ii) had at least four symptoms on the Adult ADHD Self-Report Scale six-item screener (Kessler et al., 2005; see the *ADHD traits* section below). All participants in the non-ADHD group confirmed that they did not have a formal clinical diagnosis of ADHD, were not being assessed for ADHD, and did not identify as having ADHD. They also completed the six-item screener to ensure that they did not meet the cutoff for ADHD. The groups were very closely matched on sex, age, education and socioeconomic status (SES), and as expected, significantly differed in ADHD traits (see Table 1). The final sample size provided at least 90% power to detect small-to-medium effects (*α* = 0.05; two-tailed) in the group comparisons (*d* = 0.35) and regression analyses (*f^2^* = 0.10).

**Table 1.** Demographic and clinical characteristics of the ADHD and Non-ADHD groups

Variables	ADHD^a^ (*n* = 200)	Non-ADHD^a^ (*n* = 200)	Group comparisons^b^			
			*X^2^*	*p*	*V* [95% CI]^c^	*BF_10_* ^d^
Sex (M:F)^e^	85:115	87:113	0.04	0.840	0.01 [0.00, 0.11]	0.13

			*t*	*p*	*d* [95% CI]^f^	*BF_10_* ^d^
Age	33.39 (9.29)	34.96 (13.71)	1.34	0.181	0.13 [−0.06, 0.33]	0.26
Education	5.20 (1.47)	4.95 (1.46)	−1.71	0.089	−0.17 [−0.37, 0.03]	0.45
SES	5.00 (1.45)	5.03 (1.40)	0.25	0.806	0.02 [−0.17, 0.22]	0.11
ADHD traits	51.96 (8.09)	22.33 (9.18)	−34.26	< 0.001	−3.43 [−3.73, −3.12]	4.58 x 10^116^

a Values represent means; standard deviations are shown in parentheses.

b Robust Welch t-tests are reported for age, education, SES and ADHD traits.

c Cramer’s *V* measure of effect size (at one degree of freedom, 0.10 = small, 0.30 = medium, 0.50 = large) with 95% confidence intervals shown in square brackets.

d Bayes Factor quantifying the strength of the evidence for the null compared to the alternative hypothesis (see *Bayesian Analyses* in the Supplementary Materials for more information, including conventions on Bayes Factor interpretation).

e We aimed to recruit 100 males and 100 females in each group, but this was not achieved due to the nature of online data collection.

f Cohen’s *d* measure of effect size (0.20 = small, 0.50 = medium, 0.80 = large) with 95% confidence intervals shown in square brackets.

### Measures

#### Demographic information

Participants’ age (years), sex (male, female, nonbinary), and subjective SES (rated between 1 and 10 using the MacArthur Scale of Subjective Social Status; Adler, Epel, Castellazzo, & Ickovics, 2000) were recorded. Additionally, participants reported their education level between 0 (no formal qualifications) and 8 (PhD or equivalent) using the International Standard Classification of Education (UNESCO Institute for Statistics, 2012).

#### ADHD traits

The 18-item Adult ADHD Self-Report Scale (ASRS; Kessler et al., 2005) assessed participants’ ADHD traits. The scale measures the frequency of symptoms related to inattention and hyperactivity/impulsivity using a 5-point Likert scale ranging from 0 (*‘never’*) to 4 (*‘very often’*). This produced total scores between 0 and 72, with higher scores corresponding to more ADHD traits. The first six items of the ASRS can also be used as a screening measure for ADHD, where ratings of 2 (*‘sometimes’*) or above on items 1–3 and ratings of 3 (*‘often’*) or above on items 4–6 are taken to indicate endorsement of a given symptom. Endorsement of at least 4 of the 6 symptoms is used as a threshold for probable ADHD. This screener has demonstrably good construct validity, with Kessler et al. (2005) noting a 97.9% classification accuracy for clinically diagnosed ADHD (see also, Hines, King, & Curry, 2012; Kessler et al., 2007).

#### ADHD-related psychological strengths

A list of potential ADHD-related psychological strengths was derived from Schippers et al. (2022) and included the 25 most commonly reported strengths (see Table 2). For each of the listed traits, participants indicated on a 7-point Likert scale, ranging from 1 (*‘strongly disagree’*) to 7 (*‘strongly agree’*), the extent to which they agreed it was a personal strength they possessed (i.e., **‘*something that [they] do well or best’*). Scores of 5 (*‘somewhat agree’*) or above were taken to indicate endorsement of a given trait as a strength.

#### Strengths knowledge

The 8-item Strengths Knowledge Scale (SKS; Govindji & Linley, 2007) measured participants’ awareness and recognition of their strengths, which were defined as the *‘things that [they] are able to do well or best’.* Participants answered items (e.g., *‘I am aware of my strengths’; ‘I know the things I am good at doing’*) on a 7-point Likert scale, which ranged from 1 (*‘strongly disagree’*) to 7 (*‘strongly agree’*). This generated total scores between 8 and 56 for each participant, with higher scores indicating that participants had greater knowledge of their strengths.

#### Strengths use

The 14-item Strengths Use Scale (SUS; Govindji & Linley, 2007) assessed the extent to which participants use their strengths (i.e., *‘things that [they] are able to do well or best’*) across different settings and situations. Participants responded to items (e.g., *‘I always play to my strengths’; ‘I am able to use my strengths in lots of different ways’*) on a 7-point Likert scale, which ranged from 1 (*‘strongly disagree’*) to 7 (*‘strongly agree’*). This generated total scores between 14 and 98 for each participant, with higher scores indicating a greater use of their strengths.

#### Quality of life

The abbreviated 26-item version of the WHO Quality of Life Instrument (WHOQOL-BREF; The WHOQOL Group, 1998) measured self-reported quality of life across four domains: physical health, psychological health, social relationships, and environment. Participants responded to items on 5-point Likert scales that ranged from 1 (e.g., *‘not at all’*/*‘never’*) to 5 (e.g., *‘an extreme amount’*/*‘always’*), and their average score in each domain was multiplied by 4, following the standard scoring conventions. This generated total scores between 4 and 20 for each of the four domains, with higher scores indicating a better quality of life.

#### Subjective wellbeing

Following Taylor et al. (2023) and other research (e.g., Govindji & Linley, 2007; Proctor, Maltby, & Linley, 2011), subjective wellbeing was measured as a composite of self-reported life satisfaction, positive affect, and negative affect. The 5-item Satisfaction With Life Scale (SWLS; Diener, Emmons, Larsen, & Griffin, 1985) assessed self-reported global life satisfaction. Participants responded to items using a 7-point Likert scale, ranging from 1 (**‘*strongly disagree’*) to 7 (*‘strongly agree’*), which produced scores between 5 and 35. The 20-item Positive and Negative Affect Schedule (PANAS; Watson, Clark, & Tellegen, 1988) assessed self-reported positive and negative affect using two 10-item subscales. Using a 5-point Likert scale ranging from 1 (*‘very slightly or not at all’*) to 5 (*‘extremely’*), participants indicated how much they felt each affect (e.g., enthusiastic, nervous) over the preceding week. Subscale scores ranged from 10 to 50. To calculate participants’ subjective wellbeing, standardized negative affect scores were subtracted from the sum of standardized life satisfaction and standardized positive affect scores, as in Taylor et al. (2023) and Govindji and Linley (2007). Higher scores indicated greater subjective wellbeing.

#### Mental health

The 21-item Depression, Anxiety and Stress Scale (DASS-21; Lovibond & Lovibond, 1995) assessed participants’ self-reported mental health symptoms in the preceding week using separate subscales for depression, anxiety, and stress. Participants responded to items on a 4-point Likert scale, ranging from 0 (**‘*did not apply to me at all’*) to 3 (*‘applied to me very much or most of the time’*), and their total score in each subscale was multiplied by 2, following the standard scoring conventions. This generated subscale scores ranging from 0 to 42, with higher scores indicating more mental health symptoms.

### Procedures

Ethical approval was granted by the Radboud University Medical Research Ethics Committee (METC: 2023-16686) and participants provided informed consent before taking part in the study. Participants completed the measures in a pseudo-randomized order to prevent any priming effects (in line with Taylor et al., 2023).

### Statistical analyses

Planned analyses were preregistered on the Open Science Framework (https://osf.io/3f8wn; Hoogman, Schippers, Shah, Laan, & Hargitai, 2023). We complement our Frequentist statistical analyses with Bayesian equivalent tests to quantify the strength of the evidence for the null compared to the alternative hypotheses. Analyses were conducted in R (version 4.4.2; R Core Team, 2024) except for the Bayesian analyses of covariance (ANCOVAs), which were run in JASP (version 0.18.3.0; JASP Team, 2024). The open dataset and analysis code are in the Supplementary Materials along with further information about the Bayesian Analyses and the interpretation of Bayes Factors.

## Results

### Internal consistency of self-report measures

All self-report measures showed good-to-excellent internal consistency, with comparable internal consistency in the ADHD and non-ADHD groups (see Table S1 in the Supplementary Materials). Importantly, both the SKS and the SUS, which had not previously been used in ADHD samples, showed excellent internal consistency, with *α* ≥ 0.90 and *ω* ≥ 0.92 in both groups.

### Comparison of self-reported strengths

A series of robust Welch t-tests were used to compare the ADHD and non-ADHD groups on their self-reported ADHD-related psychological strengths. We supplemented these analyses with Bayesian t-tests to quantify the evidence supporting the existence of group differences. As shown in Table 2, the ADHD group (*M* = 17.58, *SD* = 4.80) generally endorsed slightly more ADHD-related psychological strengths than the non-ADHD group (*M* = 16.33, *SD* = 4.90), although the evidence for an overall group difference was anecdotal (*BF_10_* = 2.69). The ADHD group rated themselves significantly higher than the non-ADHD group on *‘creative’*, *‘hyperfocus’*, *‘imaginative’*, *‘humor’*, *‘spontaneous’*, *‘up for anything’*, *‘seeing opportunities’*, *‘having broad interests’*, *‘image thinking’*, and *‘intuitive’* (all *BF_10_* > 3, indicating at least substantial evidence for these differences). In contrast, the non-ADHD group rated themselves significantly higher on *‘perseverant’* than the ADHD group, although the evidence for this difference was only anecdotal (*BF_10_* = 1.42). There were no significant group differences in the remaining 14 ADHD-related psychological strengths, with the Bayes Factors (*BF_10_* < 0.30) indicating at least substantial evidence for the null hypothesis across these strengths (except for *‘associative/seeing connections’* and *‘inquisitive’* for which there was only anecdotal evidence). These findings remained consistent after outliers were removed, with the exception of *‘inquisitive’*, which was rated significantly higher by the ADHD group: *t*(392.44) = −2.33, *p* = 0.021, *M_d_* [95% bootstrap CI] = −0.28 [−0.51, −0.05], *d* [95% CI] = −0.23 [−0.43, −0.04], *BF_10_* = 1.50 (see Table S9 in the Supplementary Materials). Exploratory chi-square tests of independence (see Table S2 in the Supplementary Materials) looking at the association between group membership and endorsement of ADHD-related strengths showed a similar pattern of results. Specifically, having ADHD was associated with endorsing *‘creative’*, *‘hyperfocus’*, *‘imaginative’*, *‘humor’*, *‘spontaneous’*, and *‘image thinking’* (all *p* ≤ 0.006, all *BF_10_* > 5) as personal strengths.

**Table 2.** Group means and mean differences in ADHD-related psychological strengths

Strengths	ADHD^a^	Non-ADHD^a^	Group differences^b^				
			*t*	*p*	*M_d_* [Bootstrap 95% CI]^c^	*d* [95% CI]^d^	*BF_10_* ^e^
Creative	5.08 (1.59)	4.54 (1.78)	−3.20	0.001	−0.54 [−0.87, − 0.21]	−0.32 [−0.52, −0.12]	14.98
Energetic	4.38 (1.66)	4.16 (1.46)	−1.41	0.160	−0.22 [−0.53, 0.10]	−0.14 [−0.34, 0.06]	0.29
Enthusiastic	5.09 (1.46)	4.94 (1.36)	−1.10	0.273	−0.16 [−0.44, 0.12]	−0.11 [−0.31, 0.09]	0.20
Resourceful	5.58 (1.15)	5.46 (1.14)	−1.01	0.315	−0.12 [−0.34, 0.12]	−0.10 [−0.30, 0.10]	0.18
Associative/seeing connections	5.17 (1.43)	4.90 (1.45)	−1.87	0.062	−0.27 [−0.56, 0.01]	−0.19 [−0.38, 0.01]	0.60
Empathic	5.61 (1.41)	5.63 (1.39)	0.14	0.887	0.02 [−0.26, 0.30]	0.01 [−0.18, 0.21]	0.11
Hyperfocus	4.92 (1.64)	4.17 (1.51)	−4.80	< 0.001	−0.76 [−1.07, −0.45]	−0.48 [−0.68, −0.28]	5844.25
Driven	4.79 (1.64)	4.81 (1.40)	0.16	0.870	0.03 [−0.28, 0.32]	0.02 [−0.18, 0.21]	0.11
Social	4.53 (1.74)	4.32 (1.74)	−1.18	0.240	−0.21 [−0.55, 0.14]	−0.12 [−0.31, 0.08]	0.22
Perseverant	5.04 (1.44)	5.35 (1.25)	2.30	0.022	0.31 [0.05, 0.57]	0.23 [0.03, 0.43]	1.42
Imaginative	5.35 (1.43)	4.83 (1.52)	−3.49	< 0.001	−0.52 [−0.81, −0.23]	−0.35 [−0.55, −0.15]	36.77
Flexible	5.09 (1.40)	5.15 (1.27)	0.41	0.682	0.06 [−0.21, 0.32]	0.04 [−0.16, 0.24]	0.12
Humour	5.86 (1.14)	5.42 (1.39)	−3.42	< 0.001	−0.44 [−0.69, −0.19]	−0.34 [−0.54, −0.14]	29.51
Spontaneous	4.63 (1.61)	4.08 (1.50)	−3.49	< 0.001	−0.55 [−0.85, −0.25]	−0.35 [−0.55, −0.15]	37.17
Thinking fast	5.17 (1.49)	5.04 (1.43)	−0.92	0.356	−0.14 [−0.42, 0.16]	−0.09 [−0.29, 0.10]	0.17
Sensitive	5.08 (1.63)	5.19 (1.49)	0.70	0.482	0.11 [−0.20, 0.41]	0.07 [−0.13, 0.27]	0.14
Being able to switch quickly between tasks	4.78 (1.65)	4.98 (1.31)	1.34	0.180	0.20 [−0.09, 0.50]	0.13 [−0.06, 0.33]	0.26
Inquisitive	5.60 (1.34)	5.37 (1.20)	−1.81	0.071	−0.23 [−0.48, 0.02]	−0.18 [−0.38, 0.02]	0.54
Stress resistant	3.75 (1.77)	3.93 (1.82)	1.00	0.316	0.18 [−0.18, 0.53]	0.10 [−0.10, 0.30]	0.18
Up for anything	4.84 (1.52)	4.38 (1.52)	−2.99	0.003	−0.46 [−0.75, −0.16]	−0.30 [−0.50, −0.10]	8.03
Seeing opportunities	5.17 (1.35)	4.80 (1.28)	−2.86	0.004	−0.38 [−0.63, −0.12]	−0.29 [−0.48, −0.09]	5.55
Having broad interests	5.20 (1.51)	4.80 (1.49)	−2.64	0.009	−0.40 [−0.68, −0.10]	−0.26 [−0.46, −0.07]	3.13
Image thinking	4.86 (1.46)	4.47 (1.46)	−2.67	0.008	−0.39 [−0.67, −0.10]	−0.27 [−0.46, −0.07]	3.34
Intuitive	5.58 (1.15)	5.21 (1.14)	−3.24	0.001	−0.37 [−0.59, −0.15]	−0.32 [−0.52, −0.13]	16.63
Happy	4.99 (1.41)	5.06 (1.37)	0.50	0.614	0.07 [−0.20, 0.34]	0.05 [−0.15, 0.25]	0.13
Total number of strengths endorsed^f^	17.58 (4.80)	16.33 (4.90)	−2.58	0.010	−1.25 [−2.20, −0.29]	−0.26 [−0.45, −0.06]	2.69

a Values represent means; standard deviations are shown in parentheses.

b Robust Welch t-tests are reported to address potential assumption violations.

c Mean difference between the ADHD and non-ADHD groups with 95% bootstrap confidence intervals (10,000 resamples). Bootstrapping was conducted to address potential assumption violations.

d Cohen’s *d* measure of effect size (0.20 = small, 0.50 = medium, 0.80 = large) with 95% confidence intervals shown in square brackets.

e Bayes factor quantifying the strength of the evidence for the null compared to the alternative hypothesis (see *Bayesian Analyses* in the Supplementary Materials for more information, including conventions on Bayes Factor interpretation).

f Scores of 5 (*‘somewhat agree’*) or above indicated endorsement of a given trait as a strength.

### Comparison of strengths knowledge and strengths use

Robust Welch and Bayesian t-tests showed that the ADHD group (*M* = 40.27, *SD* = 7.85) did not significantly differ in strengths knowledge from the non-ADHD group (*M* = 40.56, *SD* = 7.36): *t*(396.38) = 0.39, *p* = 0.699, *M_d_* [95% bootstrap CI] = 0.30 [−1.18, 1.79], *d* [95% CI] = 0.04 [−0.16, 0.23], *BF_10_* = 0.12. The ADHD group (*M* = 68.46, *SD* = 14.15) also did not significantly differ in strengths use from the non-ADHD group (*M* = 68.18, *SD* = 14.20): *t*(397.99) = −0.20, *p* = 0.844, *M_d_* [95% bootstrap CI] = −0.28 [−3.07, 2.48], *d* [95% CI] = −0.02 [−0.22, 0.18], *BF_10_* = 0.11. Frequentist and Bayesian ANCOVAs further showed that there was still no group difference in strengths use after controlling for strengths knowledge: *F*(1, 397) = 0.44, *p* = 0.505, *η_p_^2^* = 0.00, *BF_incl_* = 0.14. These findings remained consistent after outliers were removed (see Table S9 in the Supplementary Materials).

### Associations between strengths knowledge, strengths use, and life outcomes

The ADHD group reported significantly lower subjective wellbeing (*p* = 0.008, *BF_10_* = 3.40), significantly lower quality of life across the physical, psychological, and environmental domains (all *p* < 0.001, all *BF_10_* > 50), and significantly more mental health symptoms (all *p* < .001, all *BF_10_* > 423) than the non-ADHD group (see Table S3 in the Supplementary Materials, for robust Welch and Bayesian t-test results). These findings remained consistent after outliers were removed (see Table S10 in the Supplementary Materials).

Using multiple linear regression analyses and Bayesian ANCOVAs, we further examined the associations of ADHD group status, strengths knowledge and strengths use with these positive and negative life outcomes, while accounting for participants’ age, sex, and education level (see Table S5 in the Supplementary Materials). We also modeled interactions between ADHD group status and each of the predictors and thus report inclusion Bayes Factors (*BF_incl_*) across matched models (for more information, see *Bayesian Analyses* in the Supplementary Materials). We found that having ADHD predicted increased mental health symptoms (all *p* < 0.001, all *BF_incl_* > 2620), lower subjective wellbeing (*p* = 0.002, *BF_incl_* = 13.19), and poorer physical, psychological, and environmental quality of life (all *p* < 0.001, all *BF_incl_* > 111). Greater strength knowledge was a significant unique predictor of all life outcomes (all *p* ≤ 0.028), although the strength of the evidence for these effects were mixed with the *BF_incl_* ranging from 1.58 for social quality of life to 68964.34 for psychological quality of life.

**Figure 1.**
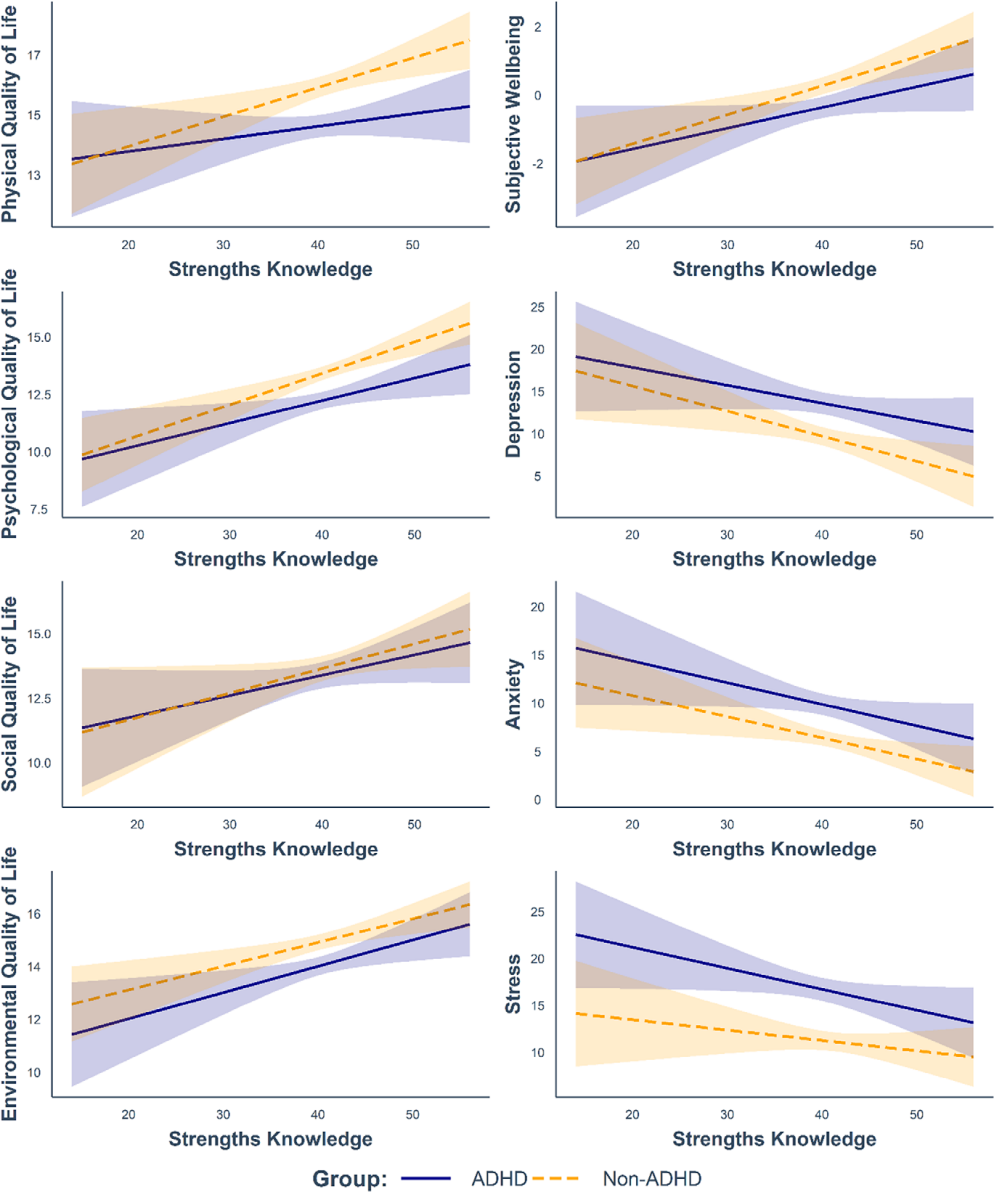
Relationships between strengths knowledge and life outcomes according to group status. *Note.* Modelled relationships are after accounting for strengths use, age, sex, and education level, as well as their interactions with ADHD. 95% confidence intervals are depicted. Results of the full moderation analyses are reported in Table S5.

**Figure 2.**
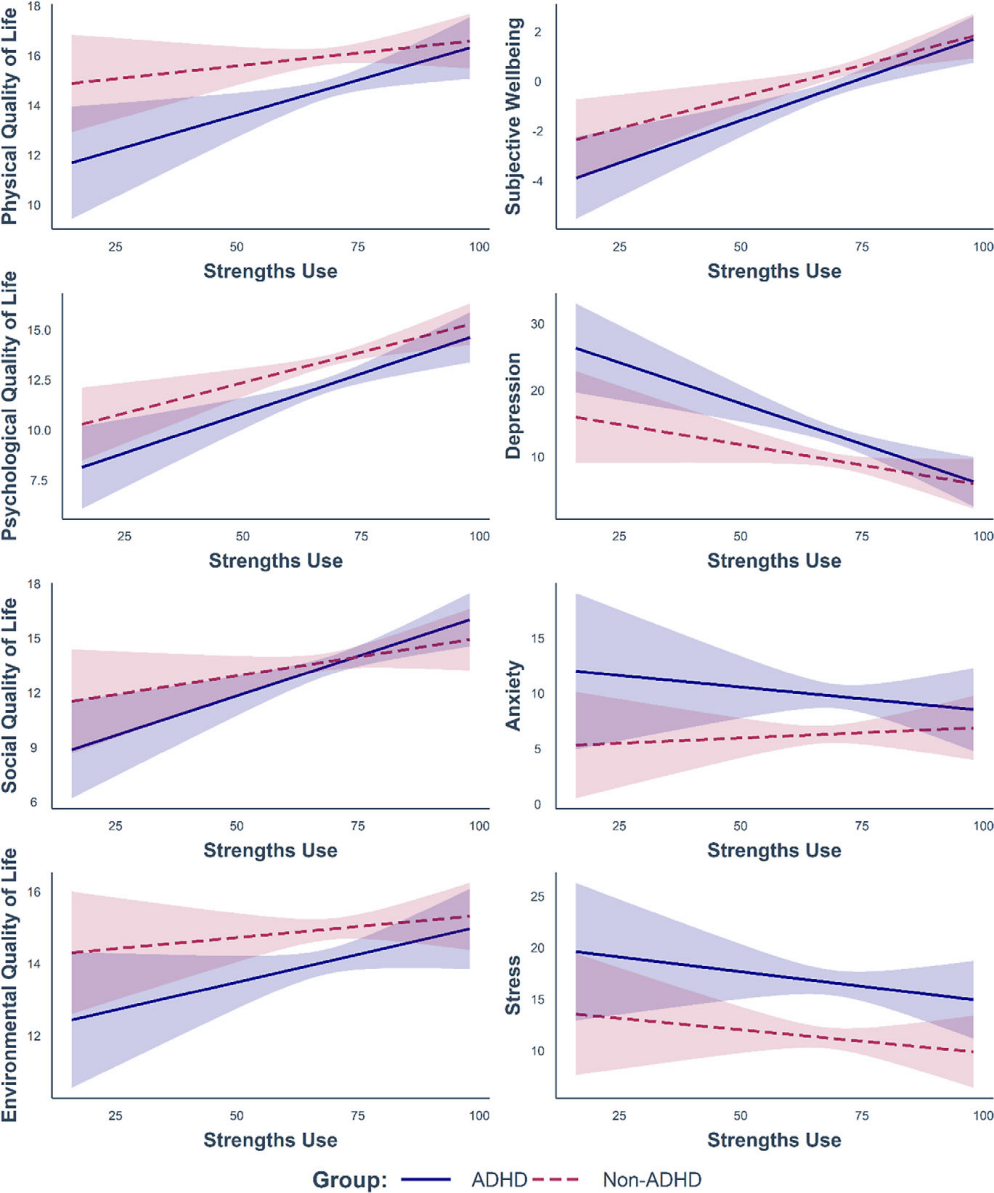
Relationships between strengths use and life outcomes according to group status. *Note.* Modelled relationships are after accounting for strengths knowledge, age, sex, and education level, as well as their interactions with ADHD. 95% confidence intervals are depicted. Results of the full moderation analyses are reported in Table S5.

Greater strengths use was found to predict higher subjective wellbeing (*p* < 0.001, *BF_incl_* = 1.95 x 10^6^), better physical, psychological, and social quality of life (all *p* ≤ 0.003, all *BF_incl_* > 14) and lower levels of depression (*p* < 0.001, *BF_incl_* = 656.58). Notably, ADHD group status did not interact with strengths knowledge (all *p* ≥ 0.241) or strengths use (all *p* ≥ 0.157) to predict any of the life outcomes, with *BF_incl_* values indicating that there was more evidence for the null hypothesis than the alternative hypothesis (see Table S5 in the Supplementary Materials). In other words, strengths knowledge and strengths use were linked to life outcomes in similar ways in the ADHD and non-ADHD groups (see Figures 1 and 2).

### Global quality of life in the ADHD group

We conducted additional regression analyses and Bayesian ANCOVAs within the ADHD group to explore predictors of global quality of life, a composite measure summing across standardized WHOQOL domain scores (see the Variable Dictionary in the Supplementary Materials for further information on how this variable was calculated). We found that strengths use was a strong positive predictor of global quality of life while controlling for participants’ strengths knowledge, sex, age, and education level (*p* < 0.001, *BF_incl_* = 1724.69; see Table S7 in the Supplementary Materials). Greater strengths knowledge also predicted better global quality of life in the ADHD group, although the evidence for this effect was weaker (*p* = 0.009, *BF_incl_* = 2.56). In a second regression model, we included ADHD traits and cross-product interactions between ADHD traits and the other predictors (see Table S8 in the Supplementary Materials). While strengths knowledge and strengths use remained significant predictors of global quality of life, there was substantial evidence that ADHD traits were not associated with this life outcome (*p* = 0.652, *BF_incl_* = 0.17). The interactions between ADHD traits and strengths knowledge and strengths use were also nonsignificant (both *p* ≥ 0.361, both *BF_incl_* ≤ 0.40). Therefore, ADHD severity did not influence the positive associations of strengths knowledge and strengths use with global quality of life in adults with ADHD.

## Discussion

This study aimed to shed new light on ADHD-related psychological strengths. Specifically, we set out to quantify these strengths and assess whether they are, indeed, more strongly endorsed by adults with ADHD compared to their neurotypical peers. We found that adults with ADHD generally endorsed more of the putative ADHD-related strengths than adults without ADHD, although our Bayesian analysis suggests that the evidence for this difference is merely anecdotal. Indeed, when examining the strengths individually, we found that adults with ADHD self-reported greater endorsement of fewer than half of the 25 listed strengths. Nonetheless, in line with the existing literature, we found that hyperfocus, creativity, and being imaginative still emerged as key ADHD-related strengths (e.g., Mahdi et al., 2017; Stolte et al., 2022; White & Shah, 2006), though creativity was less prominent than we had predicted. Spontaneity, humor, and intuitiveness emerged as additional notable strengths. The only putative ADHD-related strength that was more strongly endorsed by the non-ADHD group was being perseverant. This relative advantage for the non-ADHD group is consistent with research linking impulsivity in ADHD to lower levels of perseverance (e.g., Egan, Dawson, & Wymbs, 2017; Lopez, Dauvilliers, Jaussent, Billieux, & Bayard, 2015), and recent findings that ADHD traits are negatively correlated with perseverance in the general population (Schippers et al., 2024).

We also aimed to better understand the extent to which adults with ADHD recognize and utilize their psychological strengths in their day-to-day lives. In contrast to autistic adults, who show lower strengths knowledge and strengths use than nonautistic adults (Taylor et al., 2023), adults with ADHD did not differ from their neurotypical peers, suggesting that the two groups have a similar level of insight into their respective strengths. To understand the impact of recognizing and using one’s strengths, we also examined the link between these constructs and a range of positive and negative life outcomes. Across the whole sample and regardless of diagnostic status, having greater knowledge of personal strengths was associated with better life outcomes, including higher subjective wellbeing, greater physical, psychological, social, and environmental quality of life and fewer mental health symptoms. In contrast, greater use of personal strengths did not predict all life outcomes but was more strongly associated with better subjective wellbeing, higher physical, psychological, and social quality of life and lower levels of depression. Within the ADHD group, frequent use of personal strengths was also much more strongly linked to better global quality of life than recognizing these strengths, irrespective of participants’ ADHD severity. Together, these findings highlight that routinely drawing on and utilizing our personal strengths may be particularly important for achieving better quality of life – at least in some domains – and may also serve as a protective factor against depression. Some evidence from character strengths interventions in the general population support this idea, showing that using strengths that are most prominent in an individual increases positive affect and life satisfaction, and decreases depression (for a meta-analysis, see Schutte & Malouff, 2019). However, when looking at the broader picture, our findings suggest that knowing that we have certain skills and positive qualities at our disposal may be more beneficial to our overall wellbeing than utilizing these strengths. This idea aligns with the wider literature on self-esteem and findings that higher levels of self-esteem and self-confidence are linked to improvements in (mental) health, wellbeing, and social functioning (Henriksen, Ranøyen, Indredavik, & Stenseng, 2017; Mann, Hosman, Schaalma, & de Vries, 2004; Pedersen et al., 2024). Thus, our findings may have important implications for strengths-based psychological interventions (e.g., psychoeducational strategies to help individuals recognize their skills and behavioral reinforcement to promote the daily use of personal strengths), in both the ADHD and general populations. Crucially, these interventions may offer broader societal and economic benefits by boosting neurodivergent adults’ educational attainment (Anderson, Or, & Maguire, 2024; Lounsbury, Fisher, Levy, & Welsh, 2009), improving their performance at work (Harzer & Ruch, 2014; Rudolph, Friedrich, Koziel, & Zacher, 2025), and reducing healthcare expenditure related to co-occurring mental health conditions (e.g., depression and anxiety, see Cardoso & McHayle, 2024; McDaid et al., 2022). Despite this potential, it is important to note that our findings are correlational, and experimental evidence (e.g., examining the impact of making people’s strengths more versus less salient on self-reported wellbeing) is needed as a precursor to the development of interventions.

Our study is the largest empirical examination of strengths in ADHD and quantifies, for the first time, the self-reported strengths of a diverse sample of adults with ADHD diagnoses. Through our analyses, we were able to identify positive traits and characteristics that are more strongly endorsed by adults with ADHD compared to their neurotypical peers, which may have important implications for psychoeducation about ADHD (e.g., highlighting the positive aspects of ADHD in educational settings or in the workplace). Additionally, by ensuring that our ADHD and non-ADHD groups were well-matched on several sociodemographic factors – all of which are independently associated with wellbeing, quality of life and mental health (e.g., Cheng, Green, Wolpert, Deighton, & Furnham, 2014; Fernández-Ballesteros, Zamarrón, & Ruíz, 2001; Reiss, 2013; Soldevila-Domenech et al., 2021) – we can be more confident in the validity of our conclusions. That is, increased strengths knowledge and, to some extent, increased strengths use predict better life outcomes regardless of diagnostic status. More generally, our large, well-powered study helps to strengthen the literature on the conceptualization of ADHD in adulthood, which is largely focused on the difficulties that are associated with this neurodevelopmental condition (see Shah & Holmes, 2023; Thapar & Cooper, 2016). Nevertheless, further research is needed to address some methodological limitations.

First, while participants in the ADHD group self-reported a formal clinical diagnosis and were screened using the ASRS six-item screener (Kessler et al., 2005), this was not verified using independent diagnostic evidence and we could not directly assess participants’ level of impairment as a diagnosing clinician would do. Having this additional information about diagnostic status (e.g., in the form of anonymized diagnostic letters from a clinician) and level of impairment would strengthen the conclusions drawn from our online study. Nevertheless, adults in the ADHD group scored significantly higher on the full-scale ADHD trait measure than those in the non-ADHD group, reflecting the presence of more ADHD symptoms. Furthermore, using participants’ total ASRS score (as a proxy for ADHD impairment severity), we tested whether ADHD severity influenced the positive associations of strengths knowledge and strengths use with global quality of life in adults with ADHD. We found no such moderation effect (see Table S8 in the Supplementary Materials).

Second, our use of Prolific allowed us to efficiently collect high-quality data from a large sample and to reach individuals who may not have been able to attend in-person testing. However, this may have introduced a self-selection bias, potentially overrepresenting individuals with lower support needs, similar to laboratory-based ADHD studies. Therefore, caution is required when generalizing our findings to the broader, heterogenous adult ADHD population.

Third, although our study provides valuable insights into self-reported strengths in ADHD, it is unclear whether this translates to heightened performance in more objective measures of these skills. Recent research found that people may overestimate their attentional difficulties when self-reporting their ADHD traits as their performance on attention control tasks does not reflect this impairment (Waldren et al., 2024). In a similar way, it is possible that participants in our study overestimated the extent of their strengths, in line with a positive illusory bias that is often observed among children and adolescents with ADHD (e.g., Emeh, Mikami, & Teachman, 2018; Owens, Goldfine, Evangelista, Hoza, & Kaiser, 2007; Volz-Sidiropoulou, Boecker, & Gauggel, 2016). Thus, it is now crucial to compare adults with and without ADHD on any ADHD-related strengths identified in our study using experimental tasks to complement the existing literature on creativity and divergent thinking (e.g., Stolte et al., 2022; White & Shah, 2006). More cognitive experimental research on hyperfocus, for example, may be especially interesting to develop theories on the extent to which hyperfocus is distinguishable from focus and how this varies as a function of ADHD. Ultimately, this could allow us to better understand the level of insight that adults with ADHD have into their personal strengths, as well as contributing to the wider psychological literature on ADHD relevant concepts (i.e., attention).

Finally, in screening out any individuals who were autistic or suspected that they were autistic during our recruitment process, we may have obtained a sample that is not fully representative of the ADHD population. This is because autism and ADHD often co-occur and have overlapping symptoms (Antshel & Russo, 2019; Lai et al., 2019). Further research, which accounts for autism and other potentially relevant neurodevelopmental and mental health conditions (e.g., dyslexia, obsessive compulsive disorder), is now needed to ensure that no ADHD-related strength is missed or mischaracterized.

Overall, our findings highlight that adults with ADHD endorse certain positive traits and characteristics, such as hyperfocus, humor, and creativity, more strongly than their neurotypical peers. Crucially, however, adults with and without ADHD did not differ on their strengths knowledge, strengths use, and, in both groups, recognizing and using more personal strengths was associated with positive life outcomes. These findings may have the potential to feed into universal strengths-based interventions. While further research is required to determine whether our findings replicate with more objective measures of psychological strengths, our study offers important new evidence on the conceptualization of ADHD in adulthood.

## Supporting information

Hargitai et al. supplementary material Hargitai et al. supplementary material

## Supplementary material

The supplementary material for this article can be found at http://doi.org/10.1017/S0033291725101232.
