# Supplementary material for: The role of psychological strengths in positive life outcomes in adults with ADHD
Source: Psychol Med. 2025 Oct 6;55:e278. doi: 10.1017/S0033291725101232 (PMC12527501; doi:10.1017/S0033291725101232)
Supplement: Hargitai et al. supplementary material [file S0033291725101232sup001.docx]

**Supplementary Materials**

*The role of psychological strengths in positive life outcomes in adults with ADHD*

For the accompanying data, variable dictionary and analysis code, please see: <https://doi.org/10.17605/OSF.IO/YUW7R>

**BAYESIAN ANALYSES**

Traditional ‘Frequentist’ statistical approaches to data analysis rely on null hypothesis significance testing, which involves testing an experimental factor against a hypothesis of no effect or no relationship between variables based on a given observation (i.e., the ‘null’ hypothesis, see Pernet, 2016). Under this approach, it is possible to reject the null hypothesis, but it is difficult to find support for it as interpreting null effects using Frequentist approaches is challenging (Wagenmakers, 2007). Bayesian inference offers an alternative statistical approach that addresses this issue.

The Bayes Factor (*BF_10_*) is a continuous measure of the strength of the evidence describing the probability of obtaining the data (D) under an explicitly outlined alternative hypothesis (H_1_) relative to the probability of obtaining the data under the null hypothesis (H_0_), see *Equation 1* below.

| ${BF}_{10}= \frac{Pr\left( D \vert H_{1} \right)}{Pr\left( D \vert H_{0} \right)}$ | (1) |
| --- | --- |

For example, a *BF_10_* of 0.25 indicates that the observed data are approximately 4 times more likely under the null hypothesis (*H_0_*) than the alternative hypothesis (*H_1_*). Following commonly accepted interpretations of *BF_10_* (see *Figure S1*), this represents ‘substantial’ evidence for the null hypothesis.

**Figure S1**

*Conventions for interpreting BF_10_ and BF_incl_ values based on Wagenmakers et al. (2011).*

**
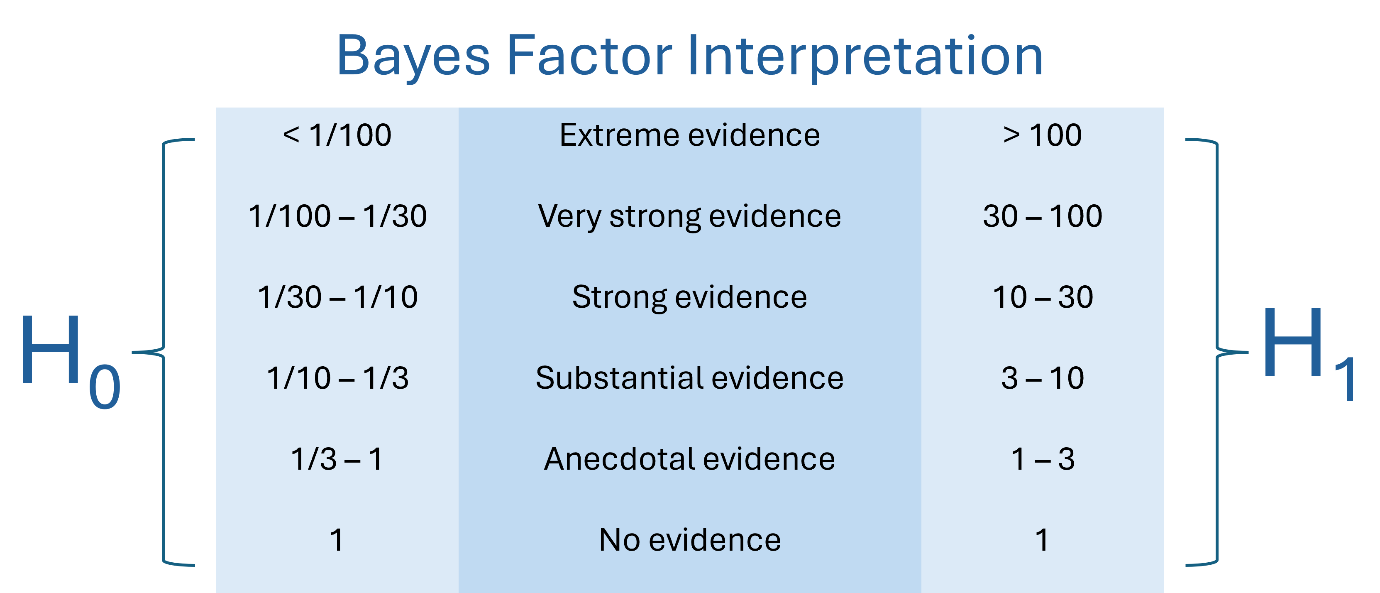
**

To complement all our Frequentist analyses, we conducted Bayesian equivalent tests to determine whether the observed data provided more evidence for the null or the alternative hypothesis. When directly comparing the ADHD and non-ADHD groups, we conducted Bayesian t-tests and used *BF_10_* to quantify the support for the two-tailed alternative hypothesis (*H_1_* = the ADHD and non-ADHD groups have significantly different mean scores) compared to the null hypothesis (*H_0_* = the ADHD and non-ADHD groups do not have significantly different mean scores). We also conducted exploratory Bayesian chi-square tests of independence to examine the association between ADHD group status and putative ADHD-related strengths, using *BF_10_* to quantify the support for the two-tailed alternative hypothesis (*H_1_* = ADHD group status is significantly associated with endorsing the given ADHD-related strength) relative to the null hypothesis (*H_0_* = ADHD group status is not significantly associated with endorsing the given ADHD-related strength). Complementing Frequentist multiple regression analyses, we used Bayesian analyses of covariance (ANCOVs) to quantify the support for the two-tailed alternative hypothesis (*H_1_* = the given predictor explains unique variance in the outcome and thus should be included in the final model) over the null hypothesis (*H_0_* = the given predictor does not explain unique variance in the outcome and thus should not be included in the final model). As we included interaction terms within these analysis models, we used inclusion Bayes Factors (*BF_incl_*) across matched models, which allowed us to compare models containing the effect compared to equivalent models without the effect, following Taylor et al. (2023). Using *BF_incl_* instead of *BF_10_* for these analyses prevents the Bayes Factors for the main effects and the interaction terms from being conflated (Mathôt, 2017).

**SENSITIVITY ANALYSES**

We conducted a series of sensitivity analyses to determine whether removing outliers influenced the results of our analyses. We used predetermined criteria to identify potential outliers. For the group comparisons, we used standardised (*z*) scores to identify univariate outliers. Any *z*-score greater than 3.29 or less than -3.29 was considered an outlier case as it belonged to the most extreme 0.1% of the reference distribution (see Mowbray et al., 2019). For the multiple regression analyses and ANCOVAs, we used the criterion of Cook’s Distance > 0.25 to identify influential datapoints.

While no datapoints were identified as multivariate outliers, several univariate outliers were detected in group comparisons. Sensitivity analyses for ADHD-related strengths, strengths knowledge and strengths use are displayed in *Table S9*, while the sensitivity analyses for positive and negative life outcomes are shown in *Table S10*.

**SUPPLEMENTARY TABLES**

| **Table S1**  *The internal consistency of measures within the ADHD and non-ADHD groups.* | | | | |
| --- | --- | --- | --- | --- |
| **Self-report Measures** | **ADHD** | | **Non-ADHD** | |
|  | ***α* ^a^** | ***ω* ^b^** | ***α* ^a^** | ***ω* ^b^** |
| Strengths Knowledge Scale | 0.91 | 0.92 | 0.90 | 0.92 |
| Strengths Use Scale | 0.93 | 0.94 | 0.94 | 0.94 |
| Adult ADHD Self-Report Scale | 0.81 | 0.81 | 0.85 | 0.86 |
| WHOQOL-BREF: Physical | 0.80 | 0.80 | 0.82 | 0.83 |
| WHOQOL-BREF: Psychological | 0.84 | 0.86 | 0.80 | 0.81 |
| WHOQOL-BREF: Social | 0.74 | 0.77 | 0.73 | 0.75 |
| WHOQOL-BREF: Environmental | 0.77 | 0.78 | 0.75 | 0.77 |
| Satisfaction With Life Scale | 0.92 | 0.92 | 0.91 | 0.92 |
| PANAS: Positive Affect | 0.91 | 0.91 | 0.91 | 0.91 |
| PANAS: Negative Affect | 0.90 | 0.90 | 0.89 | 0.89 |
| DASS-21: Depression | 0.91 | 0.91 | 0.90 | 0.90 |
| DASS-21: Anxiety | 0.85 | 0.86 | 0.72 | 0.75 |
| DASS-21: Stress | 0.85 | 0.85 | 0.83 | 0.83 |
| *Note.*  ^a^ Cronbach’s *α* quantifies the internal consistency between all items of a given scale using the correlation between items.  ^b^ McDonald’s *ω* quantifies the internal consistency between all items of a given scale using a factor analytic approach. | | | | |

| **Table S2**  *Exploratory analysis of the associations between group membership and the endorsement of ADHD-related psychological strengths* | | | | | | | |
| --- | --- | --- | --- | --- | --- | --- | --- |
| **Strength ^a^** | **ADHD ^b^** | **Non-ADHD ^b^** | ***X^2^*** | ***p*** | ***OR* [95% CI]** | ***V* [95% CI] ^c^** | ***BF_10_* ^d^** |
| Creative | 145 (72.50) | 119 (59.50) | 7.53 | 0.006 | 1.79 [1.16, 2.79] | 0.14 [0.06, 0.24] | 5.07 |
| Energetic | 107 (53.50) | 94 (47.00) | 1.69 | 0.194 | 1.30 [0.86, 1.96] | 0.07 [0.00, 0.17] | 0.29 |
| Enthusiastic | 141 (70.50) | 144 (72.00) | 0.11 | 0.740 | 0.93 [0.59, 1.47] | 0.02 [0.00, 0.12] | 0.12 |
| Resourceful | 171 (85.50) | 170 (85.00) | 0.02 | 0.888 | 1.04 [0.58, 1.88] | 0.01 [0.00, 0.10] | 0.09 |
| Associative / seeing connections | 146 (73.00) | 134 (67.00) | 1.71 | 0.190 | 1.33 [0.85, 2.10] | 0.07 [0.00, 0.17] | 0.27 |
| Empathic | 162 (81.00) | 160 (80.00) | 0.06 | 0.801 | 1.07 [0.63. 1.80] | 0.01 [0.00, 0.11] | 0.10 |
| Hyperfocus | 132 (66.00) | 85 (42.50) | 22.25 | < 0.001 | 2.63 [1.72, 4.02] | 0.24 [0.15, 0.34] | 8867.67 |
| Driven | 125 (62.50) | 120 (60.00) | 0.26 | 0.608 | 1.11 [0.73, 1.70] | 0.03 [0.00, 0.13] | 0.14 |
| Social | 117 (58.50) | 107 (53.50) | 1.01 | 0.314 | 1.23 [0.81, 1.86] | 0.05 [0.00, 0.16] | 0.21 |
| Perseverant | 139 (69.50) | 158 (79.00) | 4.72 | 0.030 | 0.61 [0.37, 0.98] | 0.11 [0.00, 0.21] | 1.15 |
| Imaginative | 159 (79.50) | 131 (65.50) | 9.83 | 0.002 | 2.04 [1.27, 3.30] | 0.16 [0.08, 0.26] | 15.24 |
| Flexible | 148 (74.00) | 151 (75.50) | 0.12 | 0.730 | 0.92 [0.57, 1.49] | 0.02 [0.00, 0.12] | 0.11 |
| Humour | 181 (90.50) | 157 (78.50) | 10.99 | < 0.001 | 2.61 [1.42, 4.94] | 0.17 [0.08, 0.27] | 23.00 |
| Spontaneous | 122 (61.00) | 93 (46.50) | 8.46 | 0.004 | 1.80 [1.19, 2.73] | 0.15 [0.07, 0.25] | 8.48 |
| Thinking fast | 144 (72.00) | 143 (71.50) | 0.01 | 0.912 | 1.02 [0.65, 1.62] | 0.01 [0.00, 0.09] | 0.11 |
| Sensitive | 141 (70.50) | 146 (73.00) | 0.31 | 0.579 | 0.88 [0.56, 1.40] | 0.03 [0.00, 0.13] | 0.13 |
| Being able to switch quickly between tasks | 127 (63.50) | 142 (71.00) | 2.55 | 0.110 | 0.71 [0.46, 1.10] | 0.08 [0.00, 0.18] | 0.42 |
| Inquisitive | 162 (81.00) | 156 (78.00) | 0.55 | 0.457 | 1.20 [0.72, 2.02] | 0.04 [0.00, 0.14] | 0.13 |
| Stress resistant | 79 (39.50) | 82 (41.00) | 0.09 | 0.760 | 0.94 [0.62, 1.43] | 0.02 [0.00, 0.12] | 0.13 |
| Up for anything | 129 (64.50) | 108 (54.00) | 4.57 | 0.033 | 1.55 [1.02, 2.36] | 0.11 [0.00, 0.21] | 1.19 |
| Seeing opportunities | 149 (74.50) | 130 (65.00) | 4.28 | 0.039 | 1.57 [1.00, 2.48] | 0.10 [0.00, 0.21] | 0.96 |
| Having broad interests | 144 (72.00) | 130 (65.00) | 2.27 | 0.132 | 1.38 [0.89, 2.17] | 0.08 [0.00, 0.18] | 0.36 |
| Image thinking | 131 (65.50) | 100 (50.00) | 9.85 | 0.002 | 1.90 [1.24, 2.90] | 0.16 [0.08, 0.26] | 16.86 |
| Intuitive | 175 (87.50) | 160 (80.00) | 4.13 | 0.042 | 1.75 [0.98, 3.15] | 0.10 [0.00, 0.21] | 0.72 |
| Happy | 139 (69.50) | 145 (72.50) | 0.44 | 0.509 | 0.86 [0.55, 1.36] | 0.03 [0.00, 0.14] | 0.14 |
| *Note.*  ^a^ Scores of 5 (*‘somewhat agree’*) or above indicated endorsement of a given trait as a strength.  ^b^ Number and percentage of participants endorsing the strengths expressed as *n* (%).  ^c^ Cramer’s *V* measure of effect size (at one degree of freedom, 0.10 = small, 0.30 = medium, 0.50 = large) with 95% confidence intervals shown in square brackets.  ^d^ Bayes Factor quantifying the strength of the evidence for the null compared to the alternative hypothesis (see *Bayesian Analyses* for more information, including conventions on Bayes Factor interpretation). | | | | | | | |

| **Table S3**  *Group means and mean differences in positive and negative life outcomes.* | | | | | | | |
| --- | --- | --- | --- | --- | --- | --- | --- |
| **Life Outcomes** | **ADHD ^a^** | **Non-ADHD ^a^** | **Group differences ^b^** | | | | |
|  |  |  | ***t*** | ***p*** | ***M_d_* [Bootstrap 95% CI] ^c^** | ***d* [95% CI] ^d^** | ***BF_10_* ^e^** |
| Subjective Wellbeing | -0.32 (2.55) | 0.32 (2.16) | 2.67 | 0.008 | 0.63 [0.17, 1.10] | 0.27 [0.07, 0.46] | 3.40 |
| Physical QOL | 14.69 (2.77) | 15.94 (2.62) | 4.63 | < 0.001 | 1.25 [0.73, 1.77] | 0.46 [0.26, 0.66] | 2774.56 |
| Psychological QOL | 12.28 (3.12) | 13.50 (2.67) | 4.21 | < 0.001 | 1.22 [0.63, 1.79] | 0.42 [0.22, 0.62] | 499.58 |
| Social QOL | 13.41 (3.93) | 13.68 (3.57) | 0.71 | 0.478 | 0.27 [-0.45, 0.99] | 0.07 [-0.13, 0.27] | 0.14 |
| Environmental QOL | 14.07 (2.61) | 14.94 (2.24) | 3.58 | < 0.001 | 0.87 [0.40, 1.36] | 0.36 [0.16, 0.56] | 50.34 |
| Depression | 13.44 (10.21) | 9.51 (8.57) | -4.17 | < 0.001 | -3.93 [-5.77, -2.09] | -0.42 [-0.61, -0.22] | 423.68 |
| Anxiety | 9.72 (7.90) | 6.22 (5.80) | -5.05 | < 0.001 | -3.50 [-4.89, -2.15] | -0.50 [-0.70, -0.31] | 18027.26 |
| Stress | 16.54 (8.77) | 11.12 (7.36) | -6.70 | < 0.001 | -5.42 [-6.99, -3.83] | -0.67 [-0.87, -0.47] | 106601107 |
| *Note.*  ^a^ Values represent means; standard deviations are shown in parentheses.  ^b^ Robust Welch t-tests are reported to address potential assumption violations.  ^c^ Mean Difference between the ADHD and non-ADHD groups with 95% bootstrap confidence intervals (10,000 resamples). Bootstrapping was conducted to address potential assumption violations.  ^d^ Cohen’s *d* measure of effect size (0.20 = small, 0.50 = medium, 0.80 = large) with 95% confidence intervals shown in square brackets.  ^e^ Bayes Factor quantifying the strength of the evidence for the null compared to the alternative hypothesis (see *Bayesian Analyses* for more information, including conventions on Bayes Factor interpretation). | | | | | | | |

| **Table S4**  *Correlations between variables.* | | | | | | | | | | | | | |
| --- | --- | --- | --- | --- | --- | --- | --- | --- | --- | --- | --- | --- | --- |
| Measures | 1 | 2 | 3 | 4 | 5 | 6 | 7 | 8 | 9 | 10 | 11 | 12 | 13 |
| 1. ADHD | – |  |  |  |  |  |  |  |  |  |  |  |  |
| 2. Strengths Knowledge | -.02 | – |  |  |  |  |  |  |  |  |  |  |  |
| 3. Strengths Use | .01 | .71*** | – |  |  |  |  |  |  |  |  |  |  |
| 4. Sex | -.02 | .04 | .02 | – |  |  |  |  |  |  |  |  |  |
| 5. Age | -.07 | .12* | -.03 | -.11* | – |  |  |  |  |  |  |  |  |
| 6. Education | .09 | .07 | .08 | -.03 | -.04 | – |  |  |  |  |  |  |  |
| 7. Subjective Wellbeing | -.13** | .48*** | .51*** | -.06 | .03 | .04 | – |  |  |  |  |  |  |
| 8. Physical QOL | -.23*** | .32*** | .33*** | .02 | -.09 | .06 | .61*** | – |  |  |  |  |  |
| 9. Psychological QOL | -.21*** | .54*** | .54*** | .03 | .05 | .01 | .85*** | .61*** | – |  |  |  |  |
| 10. Social QOL | -.04 | .33*** | .37*** | -.07 | -.03 | -.01 | .64*** | .35*** | .59*** | – |  |  |  |
| 11. Environmental QOL | -.18*** | .37*** | .33*** | .01 | -.05 | .03 | .64*** | .58*** | .60*** | .46*** | – |  |  |
| 12. Depression | .20*** | -.40*** | -.41*** | .08 | -.08 | -.05 | -.81*** | -.57*** | -.76*** | -.55*** | -.54*** | – |  |
| 13. Anxiety | .25*** | -.27*** | -.18*** | -.01 | -.11* | -.04 | -.54*** | -.52*** | -.49*** | -.32*** | -.45*** | .63*** | – |
| 14. Stress | .32*** | -.22*** | -.18*** | -.03 | -.08 | -.02 | -.61*** | -.49*** | -.56*** | -.32*** | -.43*** | .67*** | .73*** |
| *Note.* ADHD group status is coded as 1 = ADHD group, 0 = non-ADHD group. Sex is coded as 1 = male, 0 = female. * *p* < 0.05, ** *p* < 0.01,  *** *p* < 0.001 | | | | | | | | | | | | | |

| **Table S5**  *Regression analyses predicting positive and negative life outcomes across both the ADHD and non-ADHD groups.* | | | | | | |
| --- | --- | --- | --- | --- | --- | --- |
| **Predictor ^a^** | ***B* [95% CIs] ^b^** | ***SE_B_* ^b^** | ***β*** | ***p*** | ***sr^2^*** | ***BF_incl_* ^c^** |
| **Model 1: Subjective Wellbeing** | | **Overall Model Fit:** *F*(11, 388) = 16.47, *p* < 0.001, *Adjusted R^2^* = 0.30 | | | | |
| ADHD **^d^** | -0.64 [-1.04, -0.24] | 0.20 | -0.13 | .0002 | .02 | 13.19 |
| Strengths Knowledge | 0.07 [0.03, 0.11] | 0.02 | 0.23 | < 0.001 | .03 | 123.50 |
| Strengths Use | 0.06 [0.04, 0.08] | 0.01 | 0.35 | < 0.001 | .06 | 1.95 x 10^6^ |
| Sex **^e^** | -0.40 [-0.79, 0.00] | 0.21 | -0.08 | 0.052 | .01 | 0.47 |
| Age | -0.00 [-0.02, 0.02] | 0.01 | -0.02 | 0.685 | .00 | 0.12 |
| Education | 0.02 [-0.12, 0.16] | 0.07 | 0.01 | 0.799 | .00 | 0.12 |
| ADHD * Strengths Knowledge | -0.02 [-0.10, 0.05] | 0.04 | -0.04 | 0.531 | .00 | 0.12 |
| ADHD * Strengths Use | 0.02 [-0.02, 0.06] | 0.02 | 0.05 | 0.401 | .00 | 0.14 |
| ADHD * Sex | -0.20 [-1.00, 0.59] | 0.41 | -0.02 | 0.625 | .00 | 0.17 |
| ADHD * Age | -0.00 [-0.04, 0.04] | 0.02 | -0.01 | 0.797 | .00 | 0.14 |
| ADHD * Education | 0.18 [-0.10, 0.46] | 0.15 | 0.06 | 0.191 | .00 | 0.28 |
| **Model 2: Physical QOL** | | **Overall Model Fit:** *F*(11, 388) = 9.07, *p* < 0.001, *Adjusted R^2^* = 0.18 | | | | |
| ADHD **^d^** | -1.33 [-1.82, -0.82] | 0.26 | -0.24 | < 0.001 | .06 | 20086.52 |
| Strengths Knowledge | 0.07 [0.02, 0.12] | 0.02 | 0.19 | 0.004 | .02 | 7.80 |
| Strengths Use | 0.04 [0.01, 0.07] | 0.01 | 0.20 | 0.003 | .02 | 14.14 |
| Sex **^e^** | -0.13 [-0.62, 0.35] | 0.25 | -0.02 | 0.621 | .00 | 0.12 |
| Age | -0.04 [-0.06, -0.01] | 0.01 | -0.16 | 0.002 | .02 | 3.95 |
| Education | 0.11 [-0.06, 0.28] | 0.09 | 0.06 | 0.218 | .00 | 0.32 |
| ADHD * Strengths Knowledge | -0.06 [-0.15, 0.04] | 0.05 | -0.08 | 0.241 | .00 | 0.26 |
| ADHD * Strengths Use | 0.04 [-0.02, 0.09] | 0.03 | 0.09 | 0.165 | .00 | 0.29 |
| ADHD * Sex | -0.69 [-1.67, 0.30] | 0.51 | -0.06 | 0.182 | .00 | 0.29 |
| ADHD * Age | -0.04 [-0.08, 0.01] | 0.03 | -0.07 | 0.141 | .00 | 0.53 |
| ADHD * Education | 0.01 [-0.35, 0.35] | 0.18 | 0.00 | 0.976 | .00 | 0.20 |
| **Model 3: Psychological QOL** | | **Overall Model Fit:** *F*(11, 388) = 22.43, *p* < 0.001, Adjusted *R^2^* = 0.37 | | | | |
| ADHD **^d^** | -1.20 [-1.68, -0.74] | 0.24 | -0.20 | < 0.001 | .04 | 29974.15 |
| Strengths Knowledge | 0.12 [0.07, 0.16] | 0.02 | 0.30 | < 0.001 | .04 | 68964.34 |
| Strengths Use | 0.07 [0.04, 0.10] | 0.01 | 0.33 | < 0.001 | .05 | 1.89 x 10^6^ |
| Sex **^e^** | -0.02 [-0.49, 0.44] | 0.24 | -0.00 | 0.935 | .00 | 0.11 |
| Age | -0.00 [-0.02, 0.02] | 0.01 | -0.00 | 0.951 | .00 | 0.11 |
| Education | -0.04 [-0.20, 0.13] | 0.09 | -0.02 | 0.634 | .00 | 0.12 |
| ADHD * Strengths Knowledge | -0.04 [-0.14, 0.05] | 0.05 | -0.05 | 0.395 | .00 | 0.12 |
| ADHD * Strengths Use | 0.02 [-0.03, 0.07] | 0.03 | 0.04 | 0.445 | .00 | 0.11 |
| ADHD * Sex | 0.04 [-0.89, 0.96] | 0.49 | 0.00 | 0.935 | .00 | 0.16 |
| ADHD * Age | -0.01 [-0.06, 0.04] | 0.03 | -0.02 | 0.714 | .00 | 0.14 |
| ADHD * Education | 0.20 [-0.13, 0.53] | 0.17 | 0.05 | 0.212 | .00 | 0.26 |
| **Model 4: Social QOL** | | **Overall Model Fit:** *F*(11, 388) = 7.12, *p* < 0.001, *Adjusted R^2^* = 0.14 | | | | |
| ADHD **^d^** | -0.27 [-0.95, 0.40] | 0.36 | -0.04 | 0.446 | .00 | 0.15 |
| Strengths Knowledge | 0.09 [0.02, 0.15] | 0.03 | 0.18 | 0.010 | .01 | 1.58 |
| Strengths Use | 0.06 [0.03, 0.10] | 0.02 | 0.24 | < 0.001 | .03 | 357.73 |
| Sex **^e^** | -0.67 [-1.37, 0.03] | 0.37 | -0.09 | 0.062 | .01 | 0.40 |
| Age | -0.02 [-0.05, 0.01] | 0.02 | -0.07 | 0.188 | .00 | 0.22 |
| Education | -0.13 [-0.38, 0.12] | 0.13 | -0.05 | 0.268 | .00 | 0.22 |
| ADHD * Strengths Knowledge | -0.02 [-0.15, 0.10] | 0.06 | -0.02 | 0.804 | .00 | 0.23 |
| ADHD * Strengths Use | 0.05 [-0.03, 0.12] | 0.04 | 0.09 | 0.198 | .00 | 0.42 |
| ADHD * Sex | 1.01 [-0.40, 2.44] | 0.73 | 0.07 | 0.160 | .00 | 0.40 |
| ADHD * Age | -0.01 [-0.08, 0.05] | 0.03 | -0.02 | 0.713 | .00 | 0.21 |
| ADHD * Education | -0.16 [-0.66, 0.33] | 0.26 | -0.03 | 0.499 | .00 | 0.25 |
| **Model 5: Environmental QOL** | | **Overall Model Fit:** *F*(11, 388) = 8.62, *p* < 0.001, *Adjusted R^2^* = 0.17 | | | | |
| ADHD **^d^** | -0.90 [-1.35, -0.46] | 0.23 | -0.18 | < 0.001 | .03 | 111.19 |
| Strengths Knowledge | 0.09 [0.05, 0.14] | 0.02 | 0.29 | < 0.001 | . 04 | 2472.75 |
| Strengths Use | 0.02 [-0.00, 0.05] | 0.01 | 0.12 | 0.060 | .01 | 0.79 |
| Sex **^e^** | -0.10 [-0.54, 0.35] | 0.23 | -0.02 | 0.652 | .00 | 0.12 |
| Age | -0.03 [-0.05, -0.00] | 0.01 | -0.12 | 0.015 | .01 | 1.31 |
| Education | 0.03 [-0.12, 0.19] | 0.08 | 0.02 | 0.706 | .00 | 0.16 |
| ADHD * Strengths Knowledge | 0.01 [-0.08, 0.09] | 0.05 | 0.01 | 0.823 | .00 | 0.22 |
| ADHD * Strengths Use | 0.02 [-0.03, 0.07] | 0.02 | 0.05 | 0.418 | .00 | 0.36 |
| ADHD * Sex | -0.77 [-1.68, 0.13] | 0.47 | -0.08 | 0.097 | .01 | 0.37 |
| ADHD * Age | -0.03 [-0.07, 0.02] | 0.02 | -0.07 | 0.178 | .00 | 0.30 |
| ADHD * Education | -0.03 [-0.35, 0.28] | 0.16 | -0.01 | 0.840 | .00 | 0.19 |
| **Model 6: Depression** | | **Overall Model Fit:** *F*(11, 388) = 11.71, *p* < 0.001, *Adjusted R^2^* = 0.23 | | | | |
| ADHD **^d^** | 3.96 [2.29, 5.61] | 0.86 | 0.21 | < 0.001 | .04 | 2620.94 |
| Strengths Knowledge | -0.25 [-0.40, -0.09] | 0.08 | -0.20 | 0.002 | .02 | 21.36 |
| Strengths Use | -0.18 [-0.27, -0.10] | 0.04 | -0.27 | < 0.001 | .03 | 656.58 |
| Sex **^e^** | 1.93 [0.34, 3.61] | 0.85 | 0.10 | 0.028 | .01 | 0.89 |
| Age | -0.01 [-0.10, 0.08] | 0.05 | -0.01 | 0.777 | .00 | 0.24 |
| Education | -0.20 [-0.82, 0.36] | 0.31 | -0.03 | 0.497 | .00 | 0.18 |
| ADHD * Strengths Knowledge | 0.09 [-0.22, 0.41] | 0.17 | 0.03 | 0.598 | .00 | 0.18 |
| ADHD * Strengths Use | -0.12 [-0.30, 0.05] | 0.09 | -0.09 | 0.157 | .00 | 0.44 |
| ADHD * Sex | 0.10 [-3.23, 3.46] | 1.70 | 0.00 | 0.952 | .00 | 0.16 |
| ADHD * Age | 0.09 [-0.09, 0.27] | 0.09 | 0.05 | 0.263 | .00 | 0.31 |
| ADHD * Education | -0.27 [-1.45, 0.90] | 0.61 | -0.02 | 0.644 | .00 | 0.19 |
| **Model 7: Anxiety** | | **Overall Model Fit:** *F*(11, 388) = 6.22, *p* < 0.001, *Adjusted R^2^* = 0.13 | | | | |
| ADHD **^d^** | 3.48 [2.16, 4.83] | 0.69 | 0.24 | < 0.001 | .06 | 24283.67 |
| Strengths Knowledge | -0.22 [-0.35, -0.08] | 0.07 | -0.24 | < 0.001 | .03 | 684.15 |
| Strengths Use | -0.01 [-0.10, 0.06] | 0.04 | -0.02 | 0.732 | .00 | 0.17 |
| Sex **^e^** | 0.14 [-1.15, 1.40] | 0.67 | 0.01 | 0.834 | .00 | 0.12 |
| Age | -0.02 [-0.08, 0.06] | 0.04 | -0.03 | 0.591 | .00 | 0.41 |
| Education | -0.26 [-0.74, 0.19] | 0.24 | -0.05 | 0.258 | .00 | 0.27 |
| ADHD * Strengths Knowledge | -0.01 [-0.27, 0.27] | 0.14 | -0.00 | 0.969 | .00 | 0.23 |
| ADHD * Strengths Use | -0.06 [-0.23, 0.08] | 0.08 | -0.06 | 0.371 | .00 | 0.49 |
| ADHD * Sex | 1.54 [-1.07, 4.17] | 1.34 | 0.05 | 0.264 | .00 | 0.22 |
| ADHD * Age | 0.11 [-0.02, 0.25] | 0.07 | 0.09 | 0.076 | .01 | 0.76 |
| ADHD * Education | -0.31 [-1.25, 0.63] | 0.49 | -0.03 | 0.497 | .00 | 0.28 |
| **Model 8: Stress** | | **Overall Model Fit:** *F*(11, 388) = 7.05, *p* < 0.001, *Adjusted R^2^* = 0.14 | | | | |
| ADHD **^d^** | 5.42 [3.83, 7.01] | 0.81 | 0.32 | < 0.001 | .10 | 1.78 x 10^8^ |
| Strengths Knowledge | -0.17 [-0.31, -0.02] | 0.08 | -0.15 | 0.028 | .01 | 11.66 |
| Strengths Use | -0.05 [-0.13, 0.03] | 0.04 | -0.08 | 0.210 | .00 | 0.39 |
| Sex **^e^** | -0.31[-1.91, 1.23] | 0.82 | -0.02 | 0.702 | .00 | 0.13 |
| Age | -0.00 [-0.09, 0.08] | 0.04 | -0.01 | 0.918 | .00 | 0.24 |
| Education | -0.17 [-0.72, 0.37] | 0.29 | -0.03 | 0.537 | .00 | 0.21 |
| ADHD * Strengths Knowledge | -0.11 [-0.40, 0.19] | 0.15 | -0.05 | 0.454 | .00 | 0.30 |
| ADHD * Strengths Use | -0.01 [-0.18, 0.15] | 0.08 | -0.01 | 0.877 | .00 | 0.34 |
| ADHD * Sex | 0.46 [-2.68, 3.62] | 1.64 | 0.01 | 0.779 | .00 | 0.16 |
| ADHD * Age | 0.15 [-0.02, 0.32] | 0.09 | 0.10 | 0.047 | .01 | 1.26 |
| ADHD * Education | -0.43 [-1.56, 0.68] | 0.58 | -0.04 | 0.432 | .00 | 0.33 |
| *Note.*  **^a^** Predictors are mean-centred, with interaction terms calculated using the centred variables.  **^b^** 95% bias-corrected and accelerated bootstrap confidence intervals (10,000 resamples) for *B* and robust standard errors (HC3) are reported to address potential assumption violations.  **^c^** Inclusion Bayes Factors (*BF_incl_*) across matched models are reported for each predictor to quantify the evidence that it explains versus does not explain unique variance in the outcome (for further information, see *Bayesian Analyses*).  **^d^** ADHD group status is coded as 1= ADHD group, 0 = non-ADHD group.  **^e^** Sex is coded as 1 = male, 0 = female. | | | | | | |

| **Table S6**  *Correlations between variables in the ADHD group.* | | | | | | |
| --- | --- | --- | --- | --- | --- | --- |
| **Measures** | **1** | **2** | **3** | **4** | **5** | **6** |
| 1. ADHD traits | – |  |  |  |  |  |
| 2. Strengths Knowledge | -.07 | – |  |  |  |  |
| 3. Strengths Use | -.03 | .70*** | – |  |  |  |
| 4. Sex | -0.20** | .02 | .08 | – |  |  |
| 5. Age | .07 | .14* | .02 | -.19** | – |  |
| 6. Education | .05 | .10 | .09 | .07 | .01 | – |
| 7. Global QOL | -.03 | .47*** | .52*** | -.02 | -.08 | .06 |
| *Note.* Sex is coded as 1 = male, 0 = female. * *p* < 0.05, ** *p* < 0.01, *** *p* < 0.001 | | | | | | |

| **Table S7**  *Regression analysis predicting global quality of life in the ADHD group.* | | | | | | |
| --- | --- | --- | --- | --- | --- | --- |
| **Predictor** | ***B* [95% CIs] ^a^** | ***SE_B_* ^a^** | ***β*** | ***p*** | ***sr^2^*** | ***BF_incl_* ^b^** |
| Strengths Knowledge | 0.12 [0.01, 0.22] | 0.05 | 0.22 | 0.009 | .02 | 2.56 |
| Strengths Use | 0.11 [0.06, 0.16] | 0.03 | 0.37 | < 0.001 | .07 | 1724.69 |
| Sex **^c^** | -0.69 [-1.71, 0.31] | 0.52 | -0.08 | 0.185 | .01 | 0.28 |
| Age | -0.06 [-0.12, 0.00] | 0.03 | -0.13 | 0.034 | .02 | 0.79 |
| Education | 0.04 [-0.31, 0.40] | 0.19 | 0.02 | 0.795 | .00 | 0.16 |
| **Overall Model Fit:** *F*(5, 194) = 17.48, *p* < 0.001, *Adjusted R^2^* = 0.29 | | | | | | |
| *Note.*  **^a^** 95% bias-corrected and accelerated bootstrap confidence intervals (10,000 resamples) for *B* and robust standard errors (HC3) are reported to address potential assumption violations.  **^b^** Inclusion Bayes Factors (*BF_incl_*) across matched models are reported for each predictor to quantify the evidence that it explains versus does not explain unique variance in the outcome (for further information, see *Bayesian Analyses*).  **^c^** Sex is coded as 1 = male, 0 = female. | | | | | | |

| **Table S8**  *Regression analysis predicting global quality of life in the ADHD group while accounting for ADHD traits.* | | | | | | |
| --- | --- | --- | --- | --- | --- | --- |
| **Predictor ^a^** | ***B* [95% CIs] ^b^** | ***SE_B_* ^b^** | ***β*** | ***p*** | ***sr^2^*** | ***BF_incl_* ^c^** |
| ADHD Traits | 0.02 [-0.05, 0.08] | 0.04 | 0.03 | 0.652 | .00 | 0.17 |
| Strengths Knowledge | 0.13 [0.02, 0.23] | 0.06 | 0.25 | 0.005 | .03 | 2.97 |
| Strengths Use | 0.11 [0.05, 0.16] | 0.03 | 0.36 | < 0.001 | .06 | 1357.11 |
| Sex **^d^** | -0.78 [-1.82, 0.23] | 0.52 | -0.09 | 0.142 | .01 | 0.29 |
| Age | -0.05 [-0.11, 0.00] | 0.03 | -0.12 | 0.054 | .01 | 0.84 |
| Education | 0.10 [-0.27, 0.47] | 0.19 | 0.04 | 0.551 | .00 | 0.18 |
| ADHD Traits * Strengths Knowledge | -0.01 [-0.02, 0.01] | 0.01 | -0.08 | 0.361 | .00 | 0.40 |
| ADHD Traits * Strengths Use | 0.00 [-0.01, 0.01] | 0.00 | 0.02 | 0.815 | .00 | 0.26 |
| ADHD Traits * Sex | -0.14 [-0.26, -0.01] | 0.06 | -0.13 | 0.041 | .01 | 0.84 |
| ADHD Traits * Age | -0.01 [-0.02, -0.00] | 0.00 | -0.14 | 0.026 | .02 | 1.30 |
| ADHD Traits * Education | -0.00 [-0.04, 0.04] | 0.02 | -0.01 | 0.885 | .00 | 0.31 |
| **Overall Model Fit:** *F*(11, 188) = 8.92, *p* < 0.001, *Adjusted R^2^* = 0.30 | | | | | | |
| *Note.*  **^a^** Predictors are mean-centred, with interaction terms calculated using the centred variables.  **^b^** 95% bias-corrected and accelerated bootstrap confidence intervals (10,000 resamples) for *B* and robust standard errors (HC3) are reported to address potential assumption violations.  **^c^** Inclusion Bayes Factors (*BF_incl_*) across matched models are reported for each predictor to quantify the evidence that it explains versus does not explain unique variance in the outcome (for further information, see *Bayesian Analyses*).  **^d^** Sex is coded as 1 = male, 0 = female. | | | | | | |

| **Table S9**  *Group means and mean differences in ADHD-related psychological strengths, strengths knowledge and strengths use with outliers removed.* | | | | | | | |
| --- | --- | --- | --- | --- | --- | --- | --- |
| **Strengths** | **ADHD ^a^** | **Non-ADHD ^a^** | **Group differences ^b^** | | | | |
|  |  |  | ***t*** | ***p*** | ***M_d_* [Bootstrap 95% CI] ^c^** | ***d* [95% CI] ^d^** | ***BF_10_* ^e^** |
| Resourceful | 5.62 (1.06) | 5.51 (1.05) | -1.10 | 0.274 | -0.12 [-0.32, 0.09] | -0.11 [-0.31, 0.09] | 0.20 |
| Empathic | 5.61 (1.41) | 5.67 (1.32) | 0.49 | 0.626 | 0.07 [-0.20, 0.34] | 0.05 [-0.15, 0.25] | 0.12 |
| Perseverant | 5.04 (1.44) | 5.37 (1.21) | 2.50 | 0.013 | 0.33 [0.08, 0.59] | 0.25 [0.05, 0.45] | 2.19 |
| Humour | 5.94 (0.97) | 5.42 (1.39) | -4.37 | < 0.001 | -0.52 [-0.76, -0.29] | -0.44 [-0.64, -0.24] | 874.39 |
| Inquisitive | 5.66 (1.22) | 5.39 (1.16) | -2.33 | 0.021 | -0.28 [-0.51, -0.05] | -0.23 [-0.43, -0.04] | 1.50 |
| Intuitive | 5.62 (1.06) | 5.25 (1.06) | -3.51 | < 0.001 | -0.37 [-0.58, -0.17] | -0.35 [-0.55, -0.15] | 39.97 |
| Total number of strengths endorsed **^f^** | 17.58 (4.80) | 16.41 (4.77) | -2.44 | 0.015 | -1.17 [-2.10, -0.20] | -0.24 [-0.44, -0.05] | 1.92 |
| Strengths Knowledge | 40.27 (7.85) | 40.69 (7.14) | 0.57 | 0.569 | 0.43 [-1.00, 1.91] | 0.06 [-0.14, 0.25] | 0.13 |
| Strengths Use | 68.46 (14.15) | 68.44 (13.74) | -0.01 | 0.990 | -0.02 [-2.66, 2.65] | -0.00 [-0.20, 0.19] | 0.11 |
| *Note.*  **^a^** Values represent means; standard deviations are shown in parentheses.  **^b^** Robust Welch t-tests are reported to address potential assumption violations.  **^c^** Mean Difference between the ADHD and non-ADHD groups with 95% bootstrap confidence intervals (10,000 resamples). Bootstrapping was conducted to address potential assumption violations.  **^d^** Cohen’s *d* measure of effect size (0.20 = small, 0.50 = medium, 0.80 = large) with 95% confidence intervals shown in square brackets.  **^e^** Bayes Factor quantifying the strength of the evidence for the null compared to the alternative hypothesis (see *Bayesian Analyses* for more information, including conventions on Bayes Factor interpretation).  **^f^** Scores of 5 (‘*somewhat agree’*) or above indicated endorsement of a given trait as a strength. | | | | | | | |

| **Table S10**  *Group means and mean differences in positive and negative life outcomes with outliers removed.* | | | | | | | |
| --- | --- | --- | --- | --- | --- | --- | --- |
| **Strengths** | **ADHD ^a^** | **Non-ADHD ^a^** | **Group differences ^b^** | | | | |
|  |  |  | ***t*** | ***p*** | ***M_d_* [Bootstrap 95% CI] ^c^** | ***d* [95% CI] ^d^** | ***BF_10_* ^e^** |
| Physical QOL | 14.75 (2.69) | 16.05 (2.42) | 5.07 | < 0.001 | 1.30 [0.79, 1.80] | 0.51 [0.31, 0.71] | 19877.63 |
| Environmental QOL | 14.07 (2.61) | 14.98 (2.18) | 3.77 | < 0.001 | 0.91 [0.44, 1.38] | 0.38 [0.18, 0.58] | 96.95 |
| Depression | 13.44 (10.21) | 9.22 (8.11) | -4.56 | < 0.001 | -4.22 [-6.07, -2.39] | -0.46 [-0.66, -0.26] | 2057.44 |
| Anxiety | 9.41 (7.33) | 6.01 (5.44) | -5.25 | < 0.001 | -3.40 [-4.69, -2.14] | -0.53 [-0.73, -0.33] | 46376.34 |
| Stress | 16.54 (8.77) | 10.97 (7.09) | -6.97 | < 0.001 | -5.57 [-7.15, -4.01] | -0.70 [-0.90, -0.50] | 552218342 |
| *Note.*  ^a^ Values represent means; standard deviations are shown in parentheses.  ^b^ Robust Welch t-tests are reported to address potential assumption violations.  ^c^ Mean Difference between the ADHD and non-ADHD groups with 95% bootstrap confidence intervals (10,000 resamples). Bootstrapping was conducted to address potential assumption violations.  ^d^ Cohen’s *d* measure of effect size (0.20 = small, 0.50 = medium, 0.80 = large) with 95% confidence intervals shown in square brackets.  ^e^ Bayes Factor quantifying the strength of the evidence for the null compared to the alternative hypothesis (see *Bayesian Analyses* for more information, including conventions on Bayes Factor interpretation). | | | | | | | |

**SUPPLEMENTARY REFERENCES**

Mathôt, S. (2017). *Bayes like a Baws: Interpreting Bayesian Repeated Measures in JASP* [Online post]. COGSCIdotNL. https://www.cogsci.nl/blog/interpreting-bayesian-repeated-measures-in-jasp

Mowbray, F. I., Fox-Wasylyshyn, S. M., & El-Masri, M. M. (2019). Univariate Outliers: A Conceptual Overview for the Nurse Researcher. *Canadian Journal of Nursing Research*, *51*(1), 31–37. https://doi.org/10.1177/0844562118786647

Pernet, C. (2016). Null hypothesis significance testing: A short tutorial. *F1000Research*, *4*, 621. https://doi.org/10.12688/f1000research.6963.3

Taylor, E. C., Livingston, L. A., Clutterbuck, R. A., Callan, M. J., & Shah, P. (2023). Psychological strengths and well-being: Strengths use predicts quality of life, well-being and mental health in autism. *Autism*, *27*(6), 1826–1839. https://doi.org/10.1177/13623613221146440

Wagenmakers, E.-J. (2007). A practical solution to the pervasive problems ofp values. *Psychonomic Bulletin & Review*, *14*(5), 779–804. https://doi.org/10.3758/BF03194105

Wagenmakers, E.-J., Wetzels, R., Borsboom, D., & van der Maas, H. L. J. (2011). Why psychologists must change the way they analyze their data: The case of psi: Comment on Bem (2011). *Journal of Personality and Social Psychology*, *100*(3), 426–432. https://doi.org/10.1037/a0022790
